# Effects of short‐term multicomponent exercise intervention on muscle power in hospitalized older patients: A secondary analysis of a randomized clinical trial

**DOI:** 10.1002/jcsm.13375

**Published:** 2023-11-21

**Authors:** Eduardo L. Cadore, Mikel Izquierdo, Juliana Lopes Teodoro, Nicolás Martínez‐Velilla, Fabricio Zambom‐Ferraresi, Emilio Hideyuki Moriguchi, Mikel L. Sáez de Asteasu

**Affiliations:** ^1^ Exercise Research Laboratory, School of Physical Education, Physiotherapy and Dance Universidade Federal do Rio Grande do Sul Porto Alegre Brazil; ^2^ Navarrabiomed Hospital Universitario de Navarra (HUN)‐Universidad Pública de Navarra (UPNA), IdiSNA Pamplona Spain; ^3^ CIBER of Frailty and Healthy Aging (CIBERFES) Instituto de Salud Carlos III Madrid Spain; ^4^ Department of Geriatric Hospital Universitario de Navarra (HUN) Pamplona Spain; ^5^ School of Medicine Universidade Federal do Rio Grande do Sul Porto Alegre Brazil

**Keywords:** Acute hospitalization, Alternative hospital care, Disability, Multicomponent exercise, Muscle function, Power training

## Abstract

**Background:**

Bed rest during hospitalization can negatively impact functional independence and clinical status of older individuals. Strategies focused on maintaining and improving muscle function may help reverse these losses. This study investigated the effects of a short‐term multicomponent exercise intervention on maximal strength and muscle power in hospitalized older patients.

**Methods:**

This secondary analysis of a randomized clinical trial was conducted in an acute care unit in a tertiary public hospital. Ninety (39 women) older patients (mean age 87.7 ± 4.8 years) undergoing acute‐care hospitalization [median (IQR) duration 8 (1.75) and 8 (3) days for intervention and control groups, respectively]) were randomly assigned to an exercise intervention group (*n* = 44) or a control group (*n* = 46). The control group received standard care hospital including physical rehabilitation as needed. The multicomponent exercise intervention was performed for 3 consecutive days during the hospitalization, consisting of individualized power training, balance, and walking exercises. Outcomes assessed at baseline and discharge were maximal strength through 1 repetition maximum test (1RM) in the leg press and bench press exercises, and muscle power output at different loads (≤30% of 1RM and between 45% and 55% of 1RM) in the leg press exercise. Mean peak power during 10 repetitions was assessed at loads between 45% and 55% of 1RM.

**Results:**

At discharge, intervention group increased 19.2 kg (Mean Δ% = 40.4%) in leg press 1RM [95% confidence interval (CI): 12.1, 26.2 kg; *P* < 0.001] and 2.9 kg (Mean Δ% = 19.7%) in bench press 1RM (95% CI: 0.6, 5.2 kg; *P* < 0.001). The intervention group also increased peak power by 18.8 W (Mean Δ% = 69.2%) (95% CI: 8.4, 29.1 W; *P* < 0.001) and mean propulsive power by 9.3 (Mean Δ% = 26.8%) W (95% CI: 2.5, 16.1 W; *P* = 0.002) at loads ≤30% of 1RM. The intervention group also increased peak power by 39.1 W (Mean Δ% = 60.0%) (95% CI: 19.2, 59.0 W; *P* < 0.001) and mean propulsive power by 22.9 W (Mean Δ% = 64.1%) (95% CI: 11.7, 34.1 W; *P* < 0.001) at loads between 45% and 55% of 1RM. Mean peak power during the 10 repetitions improved by 20.8 W (Mean Δ% = 36.4%) (95% CI: 3.0, 38.6 W; *P* = 0.011). No significant changes were observed in the control group for any endpoint.

**Conclusions:**

An individualized multicomponent exercise program including progressive power training performed over 3 days markedly improved muscle strength and power in acutely hospitalized older patients.

## Introduction

Prolonged bed rest and consequent physical inactivity are well known causes of functional and cognitive declines during acute hospitalization in older adults, particularly in those who are physically frail.^1^, ^2^, ^3^, ^4^ These declines have severe short‐ and long‐term consequences including dependency, increased risk of falls, frequent hospital admission, disability, institutionalization, and death.^5^, ^6^, ^7^ Prolonged bed‐rest episodes during hospitalization in older adults can result in losses in skeletal muscle mass, muscle strength and muscle power output.^8^, ^9^ Indeed, losses in the muscle function (i.e., strength and power) are strongly associated with functional decline, long‐term disability and increased risk of mortality in older adults.^10^, ^11^, ^12^ Therefore, alternative care not only focusing in the illness treatment, but also on preserving physical functioning through exercise intervention has gained attention in recent years.^6^, ^13^

Multicomponent exercise intervention including resistance/power training has been shown to be an effective strategy to enhance the physical functioning in older individuals.^14^, ^15^ In a hospital setting, a multicomponent exercise intervention promoted marked improvements in the functional capacity and cognitive function in acutely hospitalized older patients.^6^, ^16^ Most of patients were responsive to the intervention and presented positive clinical changes, although a high inter‐individual variability was observed in response to in‐hospital exercise programme.^4^ Moreover, the same exercise programme induced increases in the maximal power output and maximal strength.^17^ This is especially relevant as these older patients naturally present severe age‐related strength and power declines,^18^ and the hospitalization exacerbates such aging detrimental effects on mechanical muscle function.^8^

Although the changes in muscle power output and maximal strength following a tailored exercise intervention including muscle power training have been determined in acutely hospitalized patients,^17^ there is a scarcity of data on the effects of a lower dose of exercise intervention (i.e., three sessions) on the muscle function in acutely hospitalized older adults. In the study by Sáez de Asteasu et al.,^17^ the older patients performed an intervention composed by 5 to 6 exercise days. In addition, considering that muscle power loss is one of the harmful effects of prolonged bed rest during hospitalization,^8^ it would be interesting to assess the capacity of the patients to maintain the power output during a prolonged task (i.e., fatigue resistance) following power training intervention or usual care, as reduced exercise capacity is one of the possible consequences of prolonged bed rest during hospitalization.^19^

It has been shown that there is a dose–response relationship the during resistance exercise training older individuals, meaning that there is an association between the maximal relative load achieved (i.e., % of 1RM) and the magnitude of maximal strength gains.^20^, ^21^ However, there is a lack of evidence regarding the possible association between training load and power produced during a power training intervention with the magnitude of the changes induced by the intervention in acutely hospitalized older patients.

Previously, it was shown that three consecutive sessions of tailored multicomponent exercise incorporating muscle power training improved function in acutely hospitalized patients.^22^ Given gaps in literature regarding exercise effects in this population, this secondary analysis stemming from a primary randomized trial^22^ aimed to investigate a short‐term multicomponent intervention with power training on maximal strength, muscle power output, and sustained power during a fatiguing task in acutely hospitalized older patients. Additionally, the study aimed to assess the association between the progression of exercise intensity (load and power output) and the magnitude of strength and power output gains during the intervention. Our first hypothesis was that a three‐session multicomponent exercise programme with an emphasis on muscle power training would be sufficient to improve muscle function (i.e., muscle strength and power output) in acutely hospitalized patients. Furthermore, our second hypothesis was that the magnitude of changes in muscle function would be related to the progression of exercise intensity.

## Methods

### Experimental design

This study presents a secondary analysis of a randomized controlled trial (RCT; NCT04600453)^22^ conducted in the Acute Care of the Elderly (ACE) unit of the Department of Geriatrics in a tertiary public hospital (Hospital Universitario de Navarra, Spain). Patients in this unit were admitted, in the vast majority, due to the occurrence of pulmonary diseases, heart failure and infectious diseases. In the first 48 h after hospital admission, acutely hospitalized patients who met the inclusion criteria and who agreed to participate in the study were randomly assigned to one of two groups: intervention group or control group (usual care). If the patient's condition was aggravated to the point of needing long‐term care, they were referred to a medium‐term hospital. During study participation, all patients were instructed to maintain their current activity practices. All patients or their legal representatives provided written consent. The study followed the principles of the Declaration of Helsinki and was approved by the Hospital Universitario de Navarra Research Ethics Committee (Pyto2018/7).

### Participants and randomization

Patients admitted to the ACE unit were evaluated by geriatricians. Participants included in this study were older men and women who were acutely hospitalized and that were recruited within the first 48 h of admission to ACE by a team of geriatricians. Despite the patients showing certain vulnerability, the search for participants focused on individuals who had sufficient levels of functional reserve and cognitive capacity, and were able to perform assessments and physical exercises. Initially, a trained research assistant conducted a screening interview to determine whether potentially eligible patients met the following inclusion criteria: age ≥ 75 years, Barthel index score ≥ 60 points two weeks prior to admission, and able to ambulate (with/without assistance) and to communicate with the research team. Exclusion criteria included expected length of stay *<* 6 days, very severe cognitive decline (i.e., Global Deterioration Scale score = 7), terminal illness, uncontrolled arrhythmias, acute pulmonary embolism and myocardial infarction, or extremity bone fracture in the past 3 months.

Study randomization was generated by an external statistician to the RCT who was blinded to the study design and participant involvement. The randomization sequence was generated through an online tool www.randomizer.org. After the baseline assessment was performed, the participants were randomly assigned following an unrestricted 1:1 ratio to the exercise (intervention group) and usual care (control group) groups. Considering the study design, it was not possible to blind the participants regarding the allocation in the groups; nevertheless, they were informed and requested not to discuss their randomization assignment with the assessment staff. The researchers involved in the evaluations were blinded with respect the involvement and assignment of patients to their respective groups.

### Interventions

Once the clinical manager deemed the patient to be in a stable condition and capable of participating in the study, the evaluation and intervention programme commenced. The same investigator, who was blinded to the participants' training group, conducted the assessments at admission and hospital discharge. The control group received standard hospital care with a focus on walking exercises to restore functionality when required. While the usual care group did not receive a formal exercise prescription, they underwent the same assessments as the intervention group, which occurred at the study's outset and after 3–4 days at hospital discharge.

The multicomponent exercise intervention was performed in an equipped room with training equipment located in the ACE geriatric unit. The training sessions took place at two times of the day, morning and afternoon, with the morning session being supervised by a researcher specializing in physical conditioning trained in safe patient handling techniques. The specialist provided verbal instructions and encouragement throughout the training session and documented adherence to the intervention programme in a daily register. The multicomponent exercise sessions lasted approximately 20 min, during three consecutive days of hospitalization (including weekends) and were considered completed when 90% or more of the planned exercises were successfully performed.

The prescribed exercises were based and adapted from the multicomponent physical exercise programme Vivifrail to prevent functional disability and risk of falls. The morning sessions included individualized supervised progressive resistance, balance, and gait retraining exercises. The resistance exercises were tailored to the individual's functional capacity using variable resistance training machines (Matrix; Johnson Health Tech and Exercycle S.L., BH Group) aiming at 2 to 3 sets of 8 to 10 repetitions with a load equivalent to 40% to 60% of the 1‐repetition maximum (1RM). Participants performed three exercises involving mainly lower‐limb muscles (squats rising from a chair, leg press, and bilateral knee extension) and one involving the upper‐body musculature (seated bench press). The researcher supervised the patients so that they performed the movements correctly and requested that the execution of the concentric phase of the exercises be at the maximum intentional speed possible, in order to optimize muscle power gains, while the eccentric phase was performed at a slower and controlled speed. In this study, besides absolute load (kg) and equivalent % of 1RM, we recorded the peak power and mean propulsive power in the leg press exercise with the aim of subsequently analysing the load and power progression between consecutive sessions.

In addition to resistance exercises, the intervention group also performed balance and gait exercises that gradually progressed in level of difficulty and proceeded as follows: semi‐tandem foot standing, stepping practice, line walking, walking with small obstacles, proprioceptive exercises on unstable surfaces using foam pads sequence, altering the base of support, and weight transfer from one leg to the other.

The second daily training session was unsupervised; notwithstanding, patients and their relatives were familiarized with the correct training procedures and executions before beginning the intervention. The exercises were performed with light loads (i.e., 0.5 to 1 kg anklets) and consisted of knee extension and flexion movements, hip abduction, handgrip with ball and daily walking in the corridor of the acute care unit with a duration based on the clinical physical exercise guide Vivifrail (vivifrail.com/resources/).^23^

### Endpoints

The endpoints of this study were maximal strength and muscle power, which were measured using the bilateral leg press exercise (Exercycle S.L., BH Group, Vitoria, Spain). The 1RM test involved progressively increasing the load until the participant could no longer lift any additional weight while maintaining proper technique and full range of motion. The 1RM value was determined as the maximum load that the participant could move through a complete range of motion. The participants' 1RM was determined through four to five attempts with a 3‐min recovery period between attempts. During testing, participants were verbally encouraged to always produce their maximum strength.^17^

After obtaining the 1RM values in the leg press, the participants performed the same exercise with two different loads to assess peak power and mean propulsive power: ≤30% of 1RM and between 45 and 55% of 1RM. We assessed the loads ≤30% of 1RM because these loads are more associated with low strength demand functional tasks such as gait ability, and the loads between 45% and 55% of 1RM because they are more associated with high strength demand functional tasks, such as rising from a chair.^24^ Participants were asked to perform three repetitions at maximal intended velocity in the concentric phase with each load. Peak power and mean propulsive power were recorded from the best repetition.^17^

The ability to maintain muscle power output during a set of 10 repetitions of leg press at maximal volitional intensity at loads ranging between 45% and 55% of 1RM was also used as an endpoint to measure changes in muscle fatigue.^17^ After the intervention period, these outcomes were assessed using the same absolute load. The power output outcomes were recorded by connecting a velocity transducer to the weight plates (T‐Force System, Ergotech, Murcia, Spain).

### Statistical analysis

The data were presented as Mean and 95% confidence intervals (95% CI) for the main outcomes and mean (SD) for socio‐demographic and clinical characteristics at baseline. To assess the normality and homogeneity of variance, Shapiro–Wilk and Levene tests were used, respectively. Baseline data were compared between groups using independent t‐tests or Chi‐squared. Generalized estimating equations (GEE) were used to test the main effects for group (intervention and control) and time (pre‐ and post‐intervention), as well as to test the time versus group interaction. Additionally, the progression in power training variables was also assessed using GEE (session 1 vs. session 2 vs. session 3). For both analyses, Least Significant Differences (LSD) post‐hoc tests were adopted for pairwise comparisons. The GEE method was selected for application of appropriate principles for analysing longitudinal data in the context of clinical trials. Given that the GEE was specifically developed for the analysis of paired and longitudinal data, and considering that the present study incorporates two factors (group and time), the GEE represents a well‐suited statistical test for this analysis.^25^ The significance level was set at α = 0.05. Moreover, the effect sizes (Cohen *d*) were calculated within groups as (Mean_post_ – Mean_pre_)/SD_pre_, which Mean_post_ is the mean values after intervention period, Mean_pre_ is the mean values before the intervention, and SD_pre_ is the standard deviation before the intervention. The *d* values were classified as small (0.2–0.5), moderate (0.5–0.8), or large (≥ 0.8). The statistical software used was SPSS for Mac (version 22.0; IBM, Greenville, SC).

## Results

**Figure 1:**
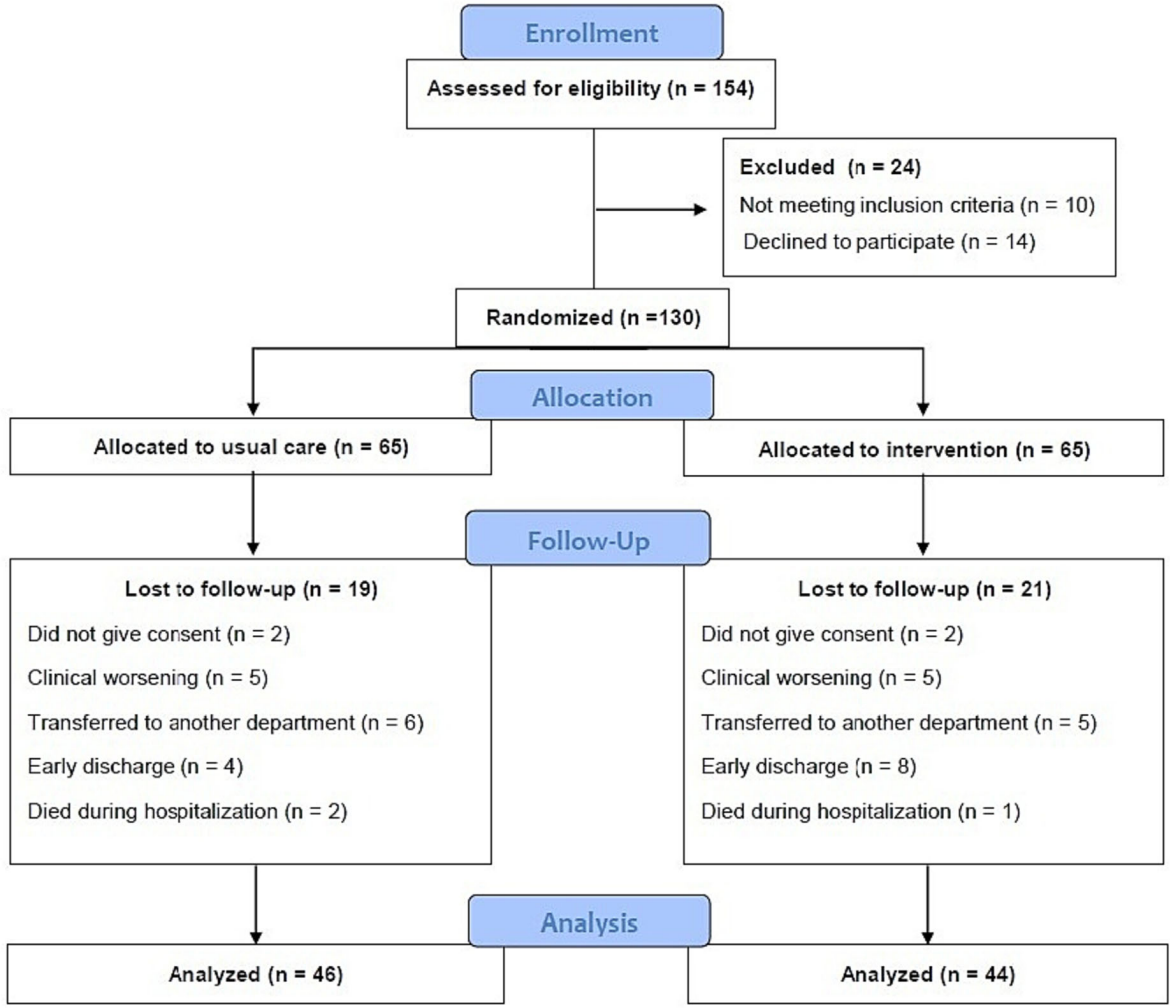
Study flow diagram.

The flowchart of study is presented in Figure 1. There were no significant differences in demographic or clinical characteristics, as well as study outcomes, between groups at baseline (Tables 1 and 3). The analyses included 90 patients, of whom 39 were women (43.3%). The mean age was 87.73 (4.84) years, with a range of 78 to 101 years, and 29 patients (32.2%) were nonagenarians. The median ± interquartile range (IQR) length of hospital stay was 8 (1.75) and 8 (3) days for intervention and control groups, respectively, with no difference between groups. The mean number of completed multicomponent exercise sessions was 3.0 with 100% of adherence to the intervention (i.e., 132 successfully completed sessions of 132 total possible sessions). At hospital admission and discharge, 74 participants performed leg‐press 1RM; 78 completed the chest‐press 1RM; 37 performed power output assessment at loads ≤30% of 1RM; 51 completed power output assessment at loads between 45% and 55% of 1RM; and 43 performed muscle power endurance test. There were no adverse effects or falls associated with the prescribed exercises, and no patients had to interrupt the intervention or change their hospital stay because of it.

**Table 1:** Baseline characteristics of the participants

	Control group (*n* = 46)	Intervention group (*n* = 44)
**Demographic data**		
Age, years	89.1 (4.7)	86.6 (4.7)
Women, *N* (%)	20 (43.5)	19 (41.3)
Body mass, kg	66.5 (14.2)	68.2 (13.9)
Height, cm	157.8 (10.0)	160.1 (9.0)
Body mass index, kg/m^2^	26.6 (4.8)	26.9 (4.8)
**Educational level**		
Illiterate, %	2.6	15.2
Primary school, %	64.1	45.6
Secondary school, %	28.2	28.3
University education, %	5.1	10.9
**Reasons for admission**		
Cardiovascular, *N* (%)	11(23.9)	11 (25.0)
Infectious, *N* (%)	16 (34.8)	17 (38.6)
Pulmonary, *N* (%)	4 (8.7)	3 (6.8%)
Gastrointestinal, *N* (%)	6 (13.0)	5 (11.4)
Neurological, *N* (%)	2 (4.3)	2 (4.5)
Others, *N* (%)	7 (15.2)	6 (13.6)
**Clinical data**		
Length of stay [median (IQR)], days	8 (3)	8 (1.75)
CIRS, score	12.8 (5.3)	12.8 (6.3)
MMSE, score	22.8 (3.9)	22.1 (5.4)
Barthel index, score	87.9 (14.8)	85.8 (16.2)
EQ‐5D, score	66.6 (22.0)	70.0 (26.7)
GDS, score	2.9 (2.5)	3.1 (2.6)
SPPB, points	4.9 (2.7)	4.9 (2.8)
GVT, s	14.5 (10.7)	13.6 (7.4)
Handgrip, kg	16.5 (5.9)	17.1 (7.4)

Data are mean (SD) otherwise indicated. No statistically significant differences were found between groups (*P* > 0.05).

CIRS, Cumulative Illness Rating Scale; EQ‐5D, Visual Analogue Scale of the EuroQol Questionnaire; GDS, Yesavage Geriatric Depression Scale; GVT, Gait Velocity Test; IQR, interquartile range; IQR, interquartile range; MMSE, Mini‐Mental State Examination; SPPB, Short Physical Performance Battery.

### Training outcomes

All recorded training outcomes showed significant relative (%) increases (*P* < 0.001) between sessions, with mean (95% CI) values as follows: absolute training load [63.9 (53.4, 74.5)]; relative training load (% of 1RM) [63.9 (53.4, 74.5)]; peak power [59 (38.5, 74.7)]; and mean propulsive power [67.9 (40.7, 95.0)] (Table 3).

### Effects of intervention

#### Overall findings

**Figure 2:**
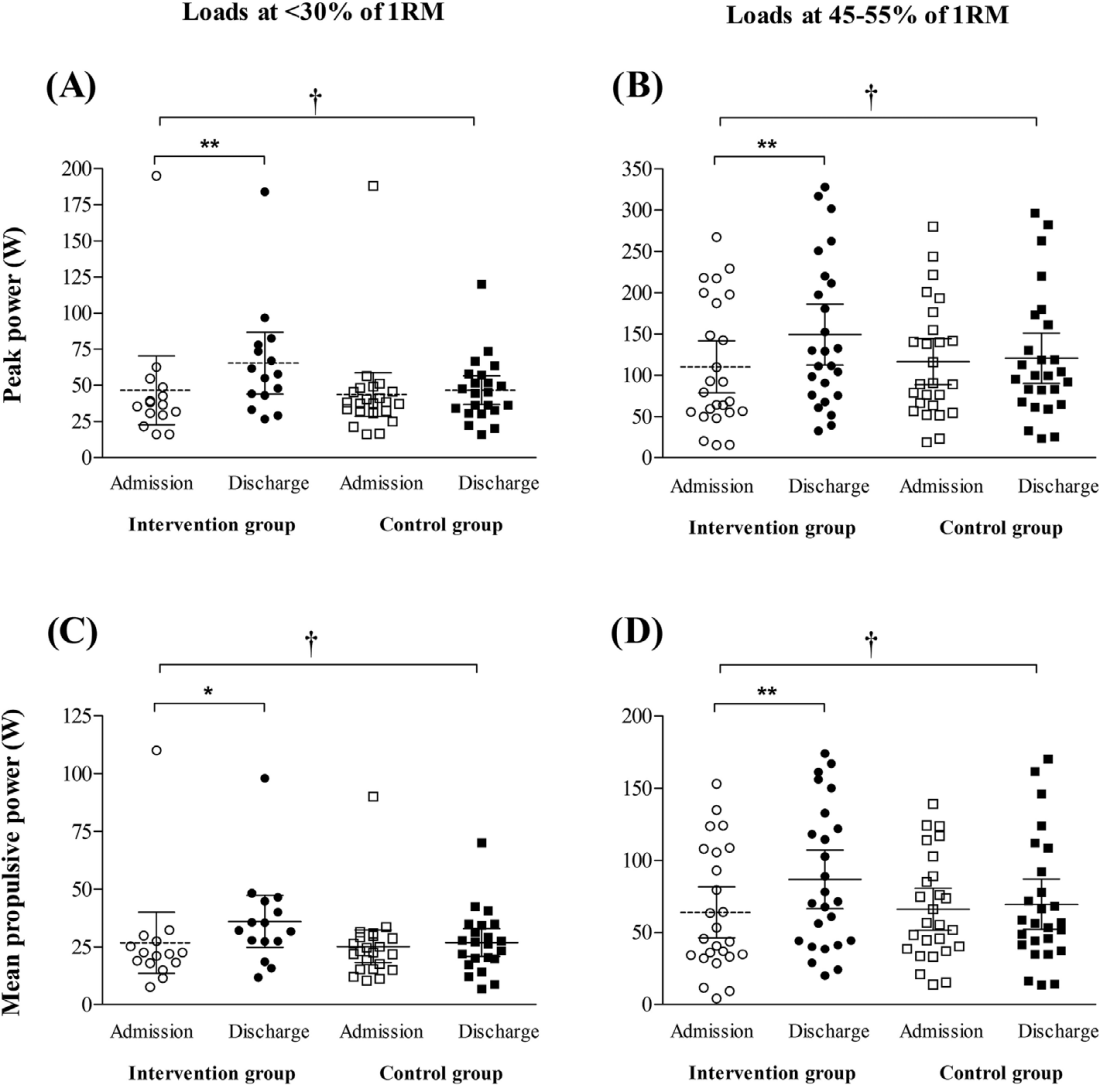
Individual values, mean and CI (95% confidence interval) from admission to hospital discharge for intervention and control groups (usual care). (A) Peak power values at loads ≤30% of 1RM; (B) peak power values at loads between 45 and 55% of 1RM; (C) mean propulsive power values at loads ≤ 30% of 1RM; (D) mean propulsive power values at loads between 45% and 55% of 1RM. Significant different from admission values: *P ≤ 0.01; **P ≤ 0.001. Significant time versus group interaction: †*P* < 0.05.

**Figure 3:**
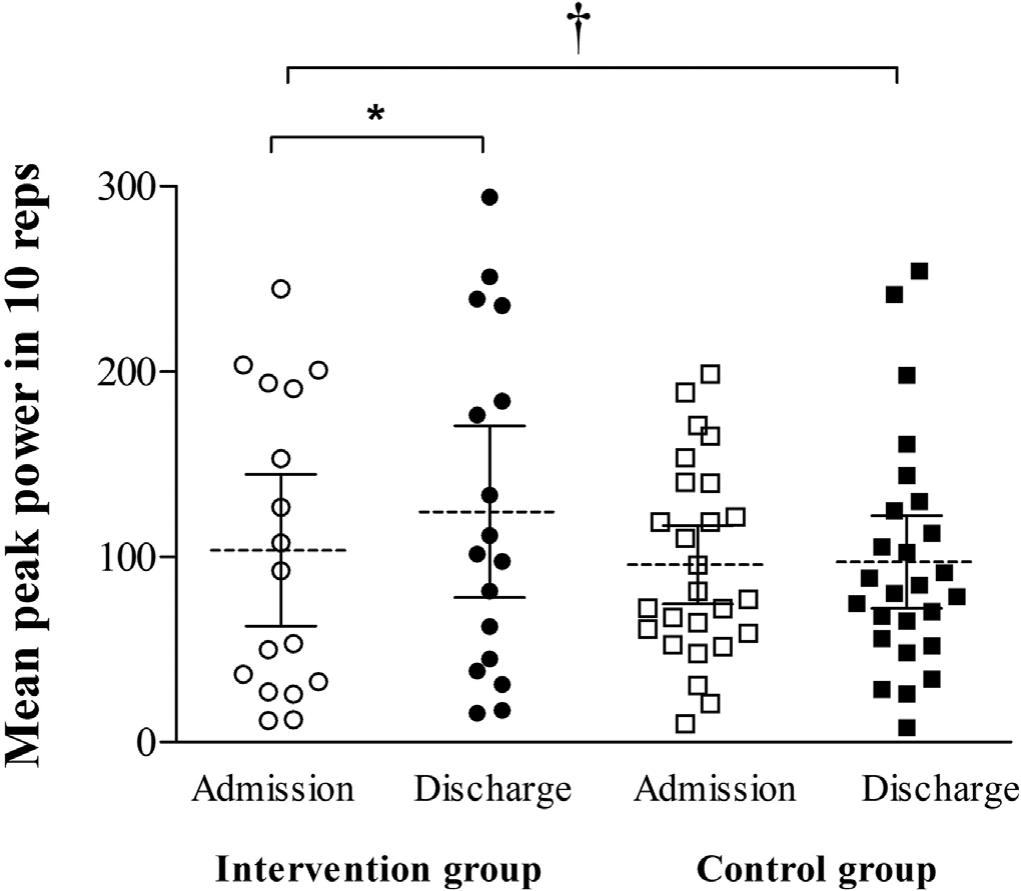
Individual values, mean and CI (95% confidence interval) from admission to hospital discharge for intervention and control groups (usual care). Significant different from admission values: *P ≤ 0.01; **P ≤ 0.001. Significant time versus group interaction: †*P* < 0.05.

Endpoints values at admission and discharge are shown in the Table 2 and Figures 2 and 3. For almost all outcomes (i.e., maximal strength, peak power, mean propulsive power, there were significant time effects (*P* values ranging from <0.001 to = 0.004) and significant time versus group interactions (*P* values ranging from <0.001 to = 0.045). Exception was observed for bench press 1RM, which there was a significant time versus group interaction (*P* < 0.001), but no significant time effect (*P* = 0.098) was observed. No significant group effects were observed in any outcome (*P* values ranging from 0.067 to 0.813).

**Table 2:** Study's outcomes at pre and post‐intervention

	Intervention group			Control group		
	Pre	Post	*d*	Pre	Post	*d*
Leg press 1RM (kg)	66.2 (53.1–79.3)	85.1 (70.6–99.5)**, †	0.46	69.4 (56.7–82.2)	73.1 (59.4–86.7)	0.09
Bench press 1RM (kg)	28.6 (25.3–31.9)	31.8 (28.7–34.9)**, †	0.30	29.1 (25.1–33.1)	27.9 (23.9–31.9)	−0.09
Loads ≤30% of 1RM						
Peak power (W)	46.6 (25.5–67.6)	65.3 (46.4–84.2)**, †	0.44	43.6 (29.8–57.5)	46.6 (37.4–55.7)	0.09
Mean propulsive power (W)	26.8 (15.1–38.5)	36.1 (26.2–46.0)*, †	0.39	25.2 (18.7–31.8)	26.9 (21.3–32.4)	0.10
Loads between 45% and 55% of 1RM						
Peak power (W)	110.1 (80.9–139.3)	149.2 (115.9–183.5)**, †	0.51	116.6 (90.4–142.8)	120.5 (92.1–148.8)	0.06
Mean propulsive power (W)	64.1 (47.6–80.6)	87.0 (68.0–105.9)**, †	0.53	66.2 (52.5–79.8)	69.6 (53.2–85.9)	0.09
Mean peak power in 10 reps	103.7 (66.8–140.5)	124.5 (82.8–165.9)*, †	0.26	95.8 (76.1–115.5)	97.4 (74.0–120.7)	0.03

Data are presented as mean, CI (95% confidence interval) and Cohen's *d* effect size.

* Significant different from pre training values (*P* ≤ 0.01).

** Significant different from pre training values (*P* ≤ 0.001).

† Significant time versus group interaction (*P* < 0.05).

**Table 3:** Power training variables during the three multicomponent exercise sessions

Variable	Session 1	Session 2	Session 3
Absolute load (kg)	29.1 (23.3–34.9)	37.0 (30.2–43.9)^**#**^	44.2 (36.7–51.8)^**#**^, ^**^
Relative load (%)	42.3 (39.9–44.6)	54.6 (51.5–57.8)^**#**^	67.5 (63.3–71.8)^**#**^, ^**^
Peak power (W)	77.2 (53.4–101.0)	95.1 (64.9–125.2)^**#**^	110.2 (77.7–142.7)^**#**^, ^*^
Mean propulsive power (W)	47.6 (33.6–61.6)	53.8 (37.3–70.4)	66.6 (49.4–83.9)^**#**^, ^**^

Data are presented as mean (95% confidence interval).

^**#**^ Significant difference from the first training session (*P* ≤ 0.001).

* Significant difference from the second training session (*P* < 0.05).

** Significant difference from the second training session (*P* < 0.001).

#### Maximal strength (1RM)

For the leg press 1RM, only the intervention group showed improvement in the leg press 1RM [mean change (95% CI) = 19.2 (12.1, 26.2), *P* < 0.001], while the usual care group showed no significant change [mean change (95% CI) = 3.6 (−4.4, 11.7), *P* = 0.349]. Regarding bench press 1RM, only the intervention group demonstrated improvement in the bench press 1RM [2.9 (0.6, 5.2), *P* = 0.001], while the usual care group showed no significant change [−0.3 (−1.7, 1.0), *P* = 0.072].

#### Muscle power output

Muscle power output was assessed at loads ≤30% and between 45% and 55% of 1RM. For lower loads (i.e., ≤30% of 1RM), only the intervention group exhibited improvement in peak power (W) [mean change (95% CI) = 18.8 (8.4, 29.1), *P* < 0.001] and mean propulsive power (W) [mean change (95% CI) = 9.3 (2.5, 16.1), *P* = 0.002]. Conversely, the usual care group did not show significant changes in these outcomes [mean change (95% CI) = 2.9 (−7.0, 12.9), and 1.7 (−3.2, 6.5) for peak power (W), and mean propulsive power (W), respectively (*P* values ranging from 0.184 to 0.528).

When evaluating muscle power outcomes at loads between 45% and 55% of 1RM, only the intervention group exhibited improvements in peak power (W) [mean change (95% CI) = 39.1 (19.2, 59.0), *P* < 0.001], and mean propulsive power (W) [mean change (95% CI) = 22.9 (11.7, 34.1), *P* < 0.001] at loads between 45% and 55% of 1RM. Conversely, no significant changes in these outcomes were observed in the usual care group [mean change (95% CI) = 3.9 (−6.8, 14.6), and 3.4 (−2.7, 9.5), for peak power (W), and mean propulsive power (W) (*P* values ranging from 0.239 to 0.719).

In terms of mean peak power (W) during the 10‐rep protocol, only the intervention group demonstrated an improvement in this outcome [mean change (95% CI) = 20.8 (3.0, 38.6), *P* = 0.011], while no significant changes were observed in the usual care group [mean change (95% CI) = 1.5 (−8.0, 11.1), *P* = 0.734).

### Associations between maximal strength gains and training variables

There were significant associations between the relative individual maximal strength gains (%) achieved by individuals and several variables. Specifically, the relative progression in training load (Rho = 0.46, *P* = 0.008), the relative progression in peak power between sessions (Rho = 0.44, *P* = 0.043), the relative progression in the mean propulsive power among sessions (Rho = 0.44, *P* = 0.039), and the maximal relative load achieved during the intervention (Rho = 0.41, *P* = 0.019) were all found to be significantly associated with individual strength gains. Furthermore, there was a significant association between the relative individual peak power gains in loads ranging from 45% to 55% of 1RM and the relative progression in peak power between sessions (Rho = 0.56, *P* = 0.017).

## Discussion

The main finding of the present study is that just three sessions of a multicomponent exercise intervention, composed of progressive power training, induced marked increases in muscle power output and maximal strength gains in older patients during acute hospitalization. Additionally, this exercise programme also improved the ability to maintain muscle power output during a set of 10 repetitions of a leg‐press exercise, suggesting a positive benefit on fatigue resistance in these patients. Furthermore, we found moderate associations between maximal strength gains and relative progression in training loading and power output during the exercise programme. Therefore, these findings contribute to our understanding of the positive effects of a multicomponent exercise programme, which minimizes the bed rest hazards associated with acute hospitalization on the neuromuscular function of older individuals.

Considering the physical inactivity phenotype, aging is associated with significant losses in maximal strength and power output, which put older individuals at greater risk of functional declines and geriatric syndromes, such as frailty, sarcopenia.^26^ This scenario is further exacerbated during acute hospitalization, as prolonged low‐mobility episodes associated with bed rest lead to an increased risk of morbidity, disability and an exacerbated decline in muscle function, particularly in very old adults.^13^, ^27^, ^28^ In the present study, we observed that even a very low volume (i.e., 3 days), but high training intensity of an individualized exercise intervention with emphasis on muscle power training improved different muscle power outcomes and maximal strength gains in older patients during acute hospitalization, whereas no changes were observed following the usual care intervention.

Our findings are consistent with those by Sáez de Asteasu et al.,^17^ who reported significant increases in lower‐limb peak power output at different loads and upper‐ and lower‐limbs maximal strength in older patients performing a median of five days of multicomponent exercise during acute hospitalization. In the present study, we applied the same exercise protocol but we tested a reduced exercise dose, which did not prevent the participants from achieving significant gains in muscle function. These findings reinforce that, in addition to adequate hospital care for acute medical disorders, a tailored exercise intervention can improve neuromuscular function in older adults during hospitalization.

In addition to peak power, we also assessed mean propulsive power to identify different mechanical adaptations following the exercise interventions. We found that all of these muscle function outcomes improved. The increase in mean propulsive power suggests that patients were able to improve power output throughout the entire acceleration phase, indicating an increased velocity during this phase, and explaining the improvement in peak power when considering the same absolute load. These changes are likely associated with concomitant enhancements in rate of force development (RFD), although RFD was not assessed in the present study.

Moreover, we observed significant improvements in muscle power output in different load ranges, including light loads (≤30% of 1RM) more associated with functional tasks with lower strength demands (i.e., gait speed), and moderate loads (between 45% and 55% of 1RM), more associated with functional tasks with high strength demands (i.e., sit‐to‐stand ability).^24^ Together with the improvement in the maximal strength, these findings indicate that only 3 days of multicomponent exercise intervention including power training can promote an overall enhancement in the mechanical muscle function in very old acutely hospitalized patients.

We also aimed to investigate the ability to maintain muscle power output during a protocol of ten leg‐press repetitions performed at near to maximal volitional intensity with the % of the 1RM. A new finding of the present study was that the intervention group improved mean peak power during this task, indicating an ability to increase their power output under fatigue conditions and produce more power during a longer task. To our best knowledge, this is the first study investigating the effects of individualized exercise intervention including power training on muscle fatigue in acutely hospitalized older patients. This finding is especially important because an increased fatigue may be one of the possible reasons for prolonged bedridden periods during hospitalization.^3^ Previous research has shown that muscle power output is significantly lost following an acute hospitalization,^8^ making our exercise intervention effective in improving the capacity to maintain power output under fatigue conditions.

Another novelty of this study was the monitoring of power progression among the three exercise sessions in the leg press exercise, which allowed us to verify the association between the magnitude of adaptations and different parameters of loading progression. We found moderate associations between maximal strength gains and different training outcomes, such as the progression in the training load, peak power and mean propulsive power, as well as the maximal relative load achieved (i.e., % of 1RM). In addition, we also observed a moderate association between relative gains in the peak power and the progression in the power output among the exercise sessions. While there is a known association between the maximal relative load achieved and the magnitude of maximal strength gains in healthy older individuals,^20^, ^29^ there is a lack of data regarding the association between training load and the magnitude of adaptations in older acutely hospitalized patients. Moreover, to our best knowledge, this is the first study investigating the association between power output progression (i.e., changes in peak power and mean propulsive power among the training sessions) and the magnitude of maximal strength and power gains in older adults, especially in a hospital setting. Although only moderate associations were observed (Rho values ranging from 0.41 to 0.56, *P* < 0.05), these findings indicate the importance of load and power progression along the exercise programme, even in a very short‐term power training intervention.

Although functional outcomes were not assessed here, muscle power output consistently associates with functional outcomes^10^, ^24^ and mortality risk^11^ in older adults. In fact, this exercise intervention previously improved Short Physical Performance Battery (SPPB) scores in hospitalized older patients.^22^ Further investigations should determine how power and strength changes translate to functional improvements in this population. Beyond muscle function, several intervention patients expressed satisfaction with the exercise regimen. Although we did not objectively assess affective outcomes, these positive comments were an encouraging qualitative aspect of the intervention.

One important consideration is that while tailored multicomponent exercise with power training effectively enhances neuromuscular function, improvements from in‐hospital interventions may not be sustained indefinitely after cessation.^5^ Training adaptations can diminish significantly with subsequent inactivity, especially in physically frail individuals.^30^ Thus, clinicians should be mindful and recommend continued exercise post‐discharge. Additionally, strategies like home‐based exercise regimens should ensure sufficient stimulus to maintain or improve physical function.

The present study has some limitations that should be mentioned. Firstly, this study is based on a secondary exploratory analysis, and the sample size was estimated according to the primary endpoints that are not discussed in this manuscript. Secondly, some patients were unable to complete all the muscle power measurements at admission and discharge, which led to the exclusion of some participants from the power output analysis. The optical encoder used to record muscle power output failed to capture some slow contraction velocities, and this could have affected the results. Finally, these findings pertain specifically to patients with a median hospital stay of 8 days and may not be applicable to patients with longer hospitalizations.

However, this study has some potential strengths that need to be highlighted. Firstly, the study was conducted on a very vulnerable population of older patients, including those with dementia and multiple comorbidities, who were hospitalized for acute illness. Secondly, the study focused on various parameters of mechanical muscle function, including different strength and power outcomes, and the power output under fatigue conditions. Lastly, the power output was monitored during the different exercise sessions to verify training progression, which is a critical factor for older individuals during acute hospitalization.

In summary, the results of this study indicate that an individualized multicomponent exercise programme that emphasizes progressive power training is an effective intervention for improving muscle power output and maximal strength gains in older patients during acute hospitalization. Furthermore, this exercise program enhances the mean muscle power during a longer protocol, which suggests a positive effect on fatigue resistance in these participants. The maximal strength and peak power gains showed a moderate association with the progression in the loads and power output throughout the program, highlighting the importance of training loading progression during the exercise program in older individuals during acute hospitalization. These findings support the use of multicomponent exercise including power training, as a crucial element to reduce the hazards of prolonged bed‐rest in older individuals, especially concerning muscle function.

## Conflict of interest

All authors of this manuscript declare no conflicts of interest.
